# Supplementary figures and images for: Design and in silico evaluation of an mRNA vaccine against HTLV-1 using AI-driven reverse vaccinology approaches
Source: PLoS One. 2026 May 6;21(5):e0340201. doi: 10.1371/journal.pone.0340201 (PMC13148667; doi:10.1371/journal.pone.0340201)

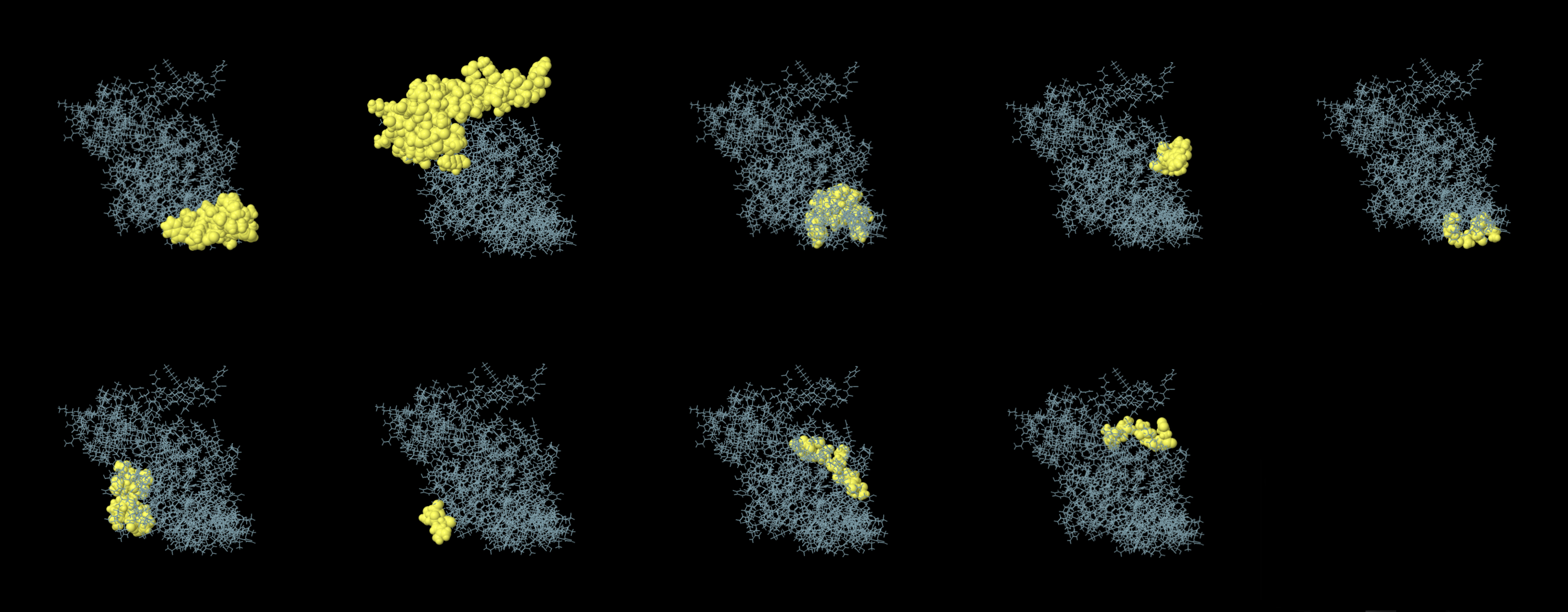

Supplement: S1 Fig — (TIFF) [file pone.0340201.s001.tiff]
