# Supplementary material for: Design and in silico evaluation of an mRNA vaccine against HTLV-1 using AI-driven reverse vaccinology approaches
Source: PLoS One. 2026 May 6;21(5):e0340201. doi: 10.1371/journal.pone.0340201 (PMC13148667; doi:10.1371/journal.pone.0340201)
Supplement: S1 Table — (DOCX) [file pone.0340201.s002.docx]

**S1 Table**. The sequences of HTLV-1 proteins.

| **Antigen** | **HTLV-1 protein’s UniPort sequence** |
| --- | --- |
| TAX | MAHFPGFGQSLLFGYPVYVFGDCVQGDWCPISGGLCSARLHRHALLATCPEHQITWDPIDGRVIGSALQFLIPRLPSFPTQRTSKTLKVLTPPITHTTPNIPPSFLQAMRKYSPFRNGYMEPTLGQHLPTLSFPDPGLRPQNLYTLWGGSVVCMYLYQLSPPITWPLLPHVIFCHPGQLGAFLTNVPYKRIEELLYKISLTTGALIILPEDCLPTTLFQPARAPVTLTAWQNGLLPFHSTLTTPGLIWTFTDGTPMISGPCPKDGQPSLVLQSSSFIFHKFQTKAYHPSFLLSHGLIQYSSFHSLHLLFEEYTNIPISLLFNEKEADDNDHEPQISPGGLEPPSEKHFRETEV |
| HBZ | MVNFVSAGLFRCLPVSCPEDLLVEELVDGLLSLEEELKDKEEEEAVLDGLLSLEEESRGRLRRGPPGEKAPPRGETHRDRQRRAEEKRKRKKEREKEEEKQTAEYLKRKEEEKARRRRRAEKKAADVARRKQEEQERRERKWRQGAEKAKQHSARKEKMQELGIDGYTRQLEGEVESLEAERRKLLQEKEDLMGEVNYWQGRLEAMWLQ |
| gp21 | CHKSLILPPFSLSPVPTLGSRSRRAVPVAVWLVSALAMGAGVAGGITGSMSLASGKSLLHEVDKDISQLTQAIVKNHKNLLKIAQYAAQNRRGLDLLFWEQGGLCKALQEQCCFLNITNSHVSILQERPPLENRVLTGWGLNWDLGLSQWAREALQTGITLVALLLLVILAGPCILR |
| Gag  (p15, p19, p24) | MGQIFPRSANPIPRPPRGLATHHWLNFLQAAYRLEPGPSSYDFHQLKTVLKMALETPVWMCPINYSLLASLLPKGYPGQVNEILQVLIQTQTQIPSHPAPPPPSSPTHDPPDSDPQIPPPYVEPTAPQVLPVMHPHGVPPTHRPWQMKDLQAIKQEVSQAAPGSPQFMQTIRLAVQQFDPTAKDLQDLLQYLCSSLVASLHHQQLDSLISEAETRGITGYNPLAGPLRVQANNPQQQGLRREYQQLWLTAFAALPGSAKDPSWASILQGLEEPYHTFVERLNVALDNGLPEGTPKDPILRSLAYSNANKECQKLLQARGHTNSPLGDMLRACQAWTPRDKTKVLVVQPKKPPPNQPCFRCGKAGHWSRDCAQPRPPPGPCPLCQDPTHWKRDCPRLKPAIPEPEPEEDALLLDLPADIPHPKNSIGGEV |
| gp62 | MGKFLATLILFFQFCPLILGDYSPSCCTLTIGVSSYHSKPCNPAQPVCSWTLDLLALSADQALQPPCPNLVSYSSYHATYSLYLFPHWIKKPNRNGGGYYSASYSDPCSLKCPYLGCQSWTCPYTGAVSSPYWKFQQDVNFTQEVSRLNINLHFSKCGFPFSLLVDAPGYDPIWFLNTEPSQLPPTAPPLLPHSNLDHILEPSIPWKSKLLTLVQLTLQSTNYTCIVCIDRASLSTWHVLYSPNVSVPSSSSTPLLYPSLALPAPHLTLPFNWTHCFDPQIQAIVSSPCHNSLILPPFSLSPVPTLGSRSRRAVPVAVWLVSALAMGAGVAGGITGSMSLASGKSLLHEVDKDISQLTQAIVKNHKNLLKIAQYAAQNRRGLDLLFWEQGGLCKALQEQCCFLNITNSHVSILQERPPLENRVLTGWGLNWDLGLSQWAREALQTGITLVALLLLVILAGPCILRQLRHLPSRVRYPHYSLINPESSL |
| p27 | MPKTRRRPRRSQRKRPPTPWAHFPGFGQSLLFGYPVYVFGDCVQGDWCPISGGLCSARLHRHALLATCPEHQITWDPIDGRVIGSALQFLIPRLPSFPTQRTSKTLKVLTPPITHTTPNIPPSFLQAMRKYSPFRNGYMEPTLGQHLPTLSFPDPGLRPQNLYTLWGGSVVCMYLYQLSPPITWPLLPHVIFCHPGQLGAFLTNVPYKRIEELLYKISLTTGALIILPEDCLPTTLFQPVKAPVTLTAWQNASFRSTQPSPLQALFGHLPMARL |
| pol | MQLAHILQPIRQAFPQCTILQYMDDILLASPSHEDLLLLSEATMASLISHGLPVSENKTQQTPGTIKFLGQIISPNHLTYDAVPTVPIRSRWALPELQALLGEIQWVSKGTPTLRQPLHSLYCALQRHTDPRDQIYLNPSQVQSLVQLRQALSQNCRSRLVQTLPLLGAIMLTLTGTTTVVFQSKQQWPLVWLHAPLPHTSQCPWGQLLASAVLLLDKYTLQSYGLLCQTIHHNISTQTFNQFIQTSDHPSVPILLHHSHRFKNLGAQTGELWNTFLKTAAPLAPVKALMPVFTLSPVIINTAPCLFSDGSTSRAAYILWDKHILSQRSFPLPPPHKSAQRAELLGLLHGLSSARSWRCLNIFLDSKYLYHYLRTLALGTFQGRSSQAPFQALLPRLLSRKVVYLHHVRSHTNLPDPISRLNALTDALLITPVLQLSPAELHSFTHCGQTALTLQGATTTEASNILRSCHACRKNNPQHQMPRGHIRRGLLPNHIWQGDITHFKYKNTLYRLHVWVDTFSGAISATQKRKETSSEAISSLLQAIAYLGKPSYINTDNGPAYISQDFLNMCTSLAIRHTTHVPYNPTSSGLVERSNGILKTLLYKYFTDKPDLPMDNALSIALWTINHLNVLTNCHKTRWQLHHSPRLQPIPETHSLSNKQTHWYYFKLPGLNSRQWKGPQEALQEAAGAALIPVSASSAQWIPWRLLKRAACPRPVGGPADPKEKDHQHHG |
| gag-pro-pol | MGQIFSRSASPIPRPPRGLAAHHWLNFLQAAYRLEPGPSSYDFHQLKKFLKIALETPARICPINYSLLASLLPKGYPGRVNEILHILIQTQAQIPSRPAPPPPSSPTHDPPDSDPQIPPPYVEPTAPQVLPVMHPHGAPPNHRPWQMKDLQAIKQEVSQAAPGSPQFMQTIRLAVQQFDPTAKDLQDLLQYLCSSLVASLHHQQLDSLISEAETRGITGYNPLAGPLRVQANNPQQQGLRREYQQLWLAAFAALPGSAKDPSWASILQGLEEPYHAFVERLNIALDNGLPEGTPKDPILRSLAYSNANKECQKLLQARGHTNSPLGDMLRACQTWTPKDKTKVLVVQPKKPPPNQPCFRCGKAGHWSRDCTQPRPPPGPCPLCQDPTHWKRDCPRLKPTIPEPEPEEDALLLDLPADIPHPKNLHRGGGLTSPPTLQQVLPNQDPASILPVIPLDPARRPVIKAQVDTQTSHPKTIEALLDTGADMTVLPIALFSSNTPLKNTSVLGAGGQTQDHFKLTSLPVLIRLPFRTTPIVLTSCLVDTKNNWAIIGRDALQQCQGVLYLPEAKRPPVILPIQAPAVLGLEHLPRPPQISQFPLNPERLQALQHLVRKALEAGHIEPYTGPGNNPVFPVKKANGTWRFIHDLRATNSLTIDLSSSSPGPPDLSSLPTTLAHLQTIDLRDAFFQIPLPKQFQPYFAFTVPQQCNYGPGTRYAWKVLPQGFKNSPTLFEMQLAHILQPIRQAFPQCTILQYMDDILLASPSHEDLLLLSEATMASLISHGLPVSENKTQQTPGTIKFLGQIISPNHLTYDAVPTVPIRSRWALPELQALLGEIQWVSKGTPTLRQPLHSLYCALQRHTDPRDQIYLNPSQVQSLVQLRQALSQNCRSRLVQTLPLLGAIMLTLTGTTTVVFQSKEQWPLVWLHAPLPHTSQCPWGQLLASAVLLLDKYTLQSYGLLCQTIHHNISTQTFNQFIQTSDHPSVPILLHHSHRFKNLGAQTGELWNTFLKTAAPLAPVKALMPVFTLSPVIINTAPCLFSDGSTSRAAYILWDKQILSQRSFPLPPPHKSAQRAELLGLLHGLSSARSWRCLNIFLDSKYLYHYLRTLALGTFQGRSSQAPFQALLPRLLSRKVVYLHHVRSHTNLPDPISRLNALTDALLITPVLQLSPAELHSFTHCGQTALTLQGATTTEASNILRSCHACRGGNPQHQMPRGHIRRGLLPNHIWQGDITHFKYKNTLYRLHVWVDTFSGAISATQKRKETSSEAISSLLQAIAHLGKPSYINTDNGPAYISQDFLNMCTSLAIRHTTHVPYNPTSSGLVERSNGILKTLLYKYFTDKPDLPMDNALSIALWTINHLNVLTNCHKTRWQLHHSPRLQPIPETRSLSNKQTHWYYFKLPGLNSRQWKGPQEALQEAAGAALIPVSASSAQWIPWRLLKRAACPRPVGGPADPKEKDLQHHG |
