# Supplementary material for: Design and in silico evaluation of an mRNA vaccine against HTLV-1 using AI-driven reverse vaccinology approaches
Source: PLoS One. 2026 May 6;21(5):e0340201. doi: 10.1371/journal.pone.0340201 (PMC13148667; doi:10.1371/journal.pone.0340201)
Supplement: S2 Table — (DOCX) [file pone.0340201.s003.docx]

**S2 Table.** Primary six suggested constructs of protein vaccine.

| Construct’s number | Order of the components | Proposed vaccine constructs |
| --- | --- | --- |
| Construct 1 | HBHA Conserved- EAAAK-PADRE-EAAAK-gp62.1- AAY-gp62.3-GPGPG-pol.1-KK-pol.2-EAAAK-RS09 | MAENSNIDDIKAPLLAALGAADLALATVNELITNLRERAEETRRSRVEESRARLTKLQEDLPEQLTELREKFTAEELRKAAEGYLEAATSELVERGEAALERLRSQQSFEEVSARAEGYVDQAVELTQEALGTVASQVEGRAAKLVGIELEAAAK**AKFVAAWTLKAAA**EAAAKWTLDLLALSADQALQPPCPNLVSYSSYHATYSLYLFPHWIKKAAYGSMSLASGKSLLHEVDKDISQLTQAIVKNHKNLLKIAQYAAQNRRGLDLLFWEQGGLCKALQEQCCFLNITNSHVSILQERGPGPGWRCLNIFLDSKYLYHYLRTLALGTFQGRSSQAPFQALLPRLLSRKVVYLHHKKAISATQKRKETSSEAISSLLQAIAYLGKPSYINTDNGPAYISQDFLNMCTSLAIRHTTHEAAAKAPPHALS |
| Construct 2 | HBHA Conserved- EAAAK-PADRE-EAAAK-gp62.1- AAY-gp62.3-GPGPG-pol.1-KK-pol.2-EAAAK-RS01 | MAENSNIDDIKAPLLAALGAADLALATVNELITNLRERAEETRRSRVEESRARLTKLQEDLPEQLTELREKFTAEELRKAAEGYLEAATSELVERGEAALERLRSQQSFEEVSARAEGYVDQAVELTQEALGTVASQVEGRAAKLVGIELEAAAK**AKFVAAWTLKAAA**EAAAKWTLDLLALSADQALQPPCPNLVSYSSYHATYSLYLFPHWIKKAAYGSMSLASGKSLLHEVDKDISQLTQAIVKNHKNLLKIAQYAAQNRRGLDLLFWEQGGLCKALQEQCCFLNITNSHVSILQERGPGPGWRCLNIFLDSKYLYHYLRTLALGTFQGRSSQAPFQALLPRLLSRKVVYLHHKKAISATQKRKETSSEAISSLLQAIAYLGKPSYINTDNGPAYISQDFLNMCTSLAIRHTTHEAAAKQEINSSY |
| Construct 3 | RS09-EAAAK-PADRE-EAAAK-gp62.1-AAY-gp62.3-GPGPG-pol.1-KK-pol.2-EAAAK-HBHA Conserved | APPHALSEAAAK**AKFVAAWTLKAAA**EAAAKWTLDLLALSADQALQPPCPNLVSYSSYHATYSLYLFPHWIKKAAYGSMSLASGKSLLHEVDKDISQLTQAIVKNHKNLLKIAQYAAQNRRGLDLLFWEQGGLCKALQEQCCFLNITNSHVSILQERGPGPGWRCLNIFLDSKYLYHYLRTLALGTFQGRSSQAPFQALLPRLLSRKVVYLHHKKAISATQKRKETSSEAISSLLQAIAYLGKPSYINTDNGPAYISQDFLNMCTSLAIRHTTHEAAAKMAENSNIDDIKAPLLAALGAADLALATVNELITNLRERAEETRRSRVEESRARLTKLQEDLPEQLTELREKFTAEELRKAAEGYLEAATSELVERGEAALERLRSQQSFEEVSARAEGYVDQAVELTQEALGTVASQVEGRAAKLVGIEL |
| Construct 4 | HBHA Conserved- EAAAK-PADRE-EAAAK-gp62.1- AAA-gp62.3-GGGS-pol.1-KK-pol.2-EAAAK-RS09 | MAENSNIDDIKAPLLAALGAADLALATVNELITNLRERAEETRRSRVEESRARLTKLQEDLPEQLTELREKFTAEELRKAAEGYLEAATSELVERGEAALERLRSQQSFEEVSARAEGYVDQAVELTQEALGTVASQVEGRAAKLVGIELEAAAK**AKFVAAWTLKAAA**EAAAKWTLDLLALSADQALQPPCPNLVSYSSYHATYSLYLFPHWIKKAAAGSMSLASGKSLLHEVDKDISQLTQAIVKNHKNLLKIAQYAAQNRRGLDLLFWEQGGLCKALQEQCCFLNITNSHVSILQERGGGSWRCLNIFLDSKYLYHYLRTLALGTFQGRSSQAPFQALLPRLLSRKVVYLHHKKAISATQKRKETSSEAISSLLQAIAYLGKPSYINTDNGPAYISQDFLNMCTSLAIRHTTHEAAAKAPPHALS |
| Construct 5 | RS09-EAAAK-PADRE-EAAAK-gp62.1-AAY-gp62.3-GPGPG--pol.1-KK-pol.2-EAAAK-RS01 | APPHALSEAAAK**AKFVAAWTLKAAA**EAAAKWTLDLLALSADQALQPPCPNLVSYSSYHATYSLYLFPHWIKKAAYGSMSLASGKSLLHEVDKDISQLTQAIVKNHKNLLKIAQYAAQNRRGLDLLFWEQGGLCKALQEQCCFLNITNSHVSILQERGPGPGWRCLNIFLDSKYLYHYLRTLALGTFQGRSSQAPFQALLPRLLSRKVVYLHHKKAISATQKRKETSSEAISSLLQAIAYLGKPSYINTDNGPAYISQDFLNMCTSLAIRHTTHEAAAKQEINSSY |
| Construct 6 | HBHA Conserved- EAAAK-PADRE-EAAAK-pol.2- AAA-gp62.3-GGGS--pol.1-KK- gp62.1-EAAAK-RS09 | MAENSNIDDIKAPLLAALGAADLALATVNELITNLRERAEETRRSRVEESRARLTKLQEDLPEQLTELREKFTAEELRKAAEGYLEAATSELVERGEAALERLRSQQSFEEVSARAEGYVDQAVELTQEALGTVASQVEGRAAKLVGIELEAAAK**AKFVAAWTLKAAA**EAAAKAISATQKRKETSSEAISSLLQAIAYLGKPSYINTDNGPAYISQDFLNMCTSLAIRHTTHAAAGSMSLASGKSLLHEVDKDISQLTQAIVKNHKNLLKIAQYAAQNRRGLDLLFWEQGGLCKALQEQCCFLNITNSHVSILQERGGGSWRCLNIFLDSKYLYHYLRTLALGTFQGRSSQAPFQALLPRLLSRKVVYLHHKKWTLDLLALSADQALQPPCPNLVSYSSYHATYSLYLFPHWIKKEAAAKAPPHALS |
