# Supplementary material for: Design and in silico evaluation of an mRNA vaccine against HTLV-1 using AI-driven reverse vaccinology approaches
Source: PLoS One. 2026 May 6;21(5):e0340201. doi: 10.1371/journal.pone.0340201 (PMC13148667; doi:10.1371/journal.pone.0340201)
Supplement: S3 Table — (DOCX) [file pone.0340201.s004.docx]

**S3 Table.** The evaluation results of 3D structures for six proposed 3D models.

| Models | | Prosa web | ERRAT (Overall Quality Factor) | Ramachandran plot |
| --- | --- | --- | --- | --- |
| Sequence 1 | | | | |
| I-TASSER | Model 1 | Z-Score: -5.97  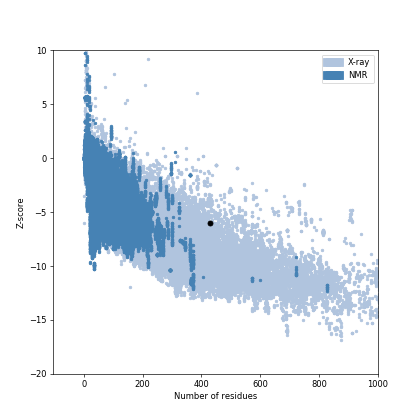 | 89.4231 | Residues in most favored regions: 81.2%  Residues in additional allowed regions: 14.2%  Residues in generously allowed regions: 3.1%  Residues in disallowed regions: 1.5% |
|  | Model 2 | Z-Score: -5.74  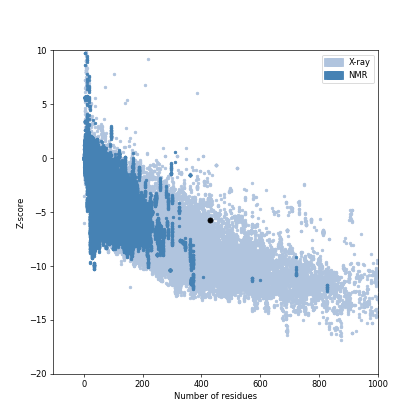 | 95.9524 | Residues in most favored regions: 85.0%  Residues in additional allowed regions: 11.2%  Residues in generously allowed regions: 1.8%  Residues in disallowed regions: 2.0% |
|  | Model 3 | Z-Score: -2.78  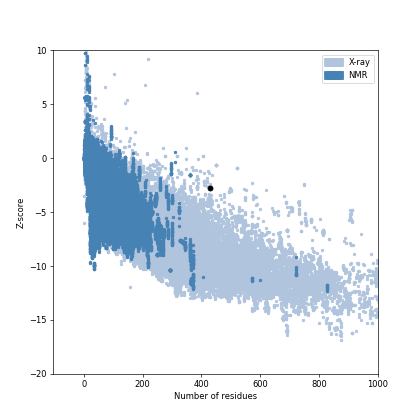 | 80.4762 | Residues in most favored regions: 71.0%  Residues in additional allowed regions: 18.1%  Residues in generously allowed regions: 5.3%  Residues in disallowed regions: 5.6% |
|  | Model 4 | Z-Score: -3.41  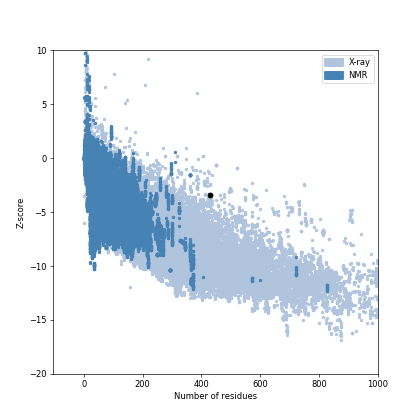 | 85.7143 | Residues in most favored regions: 76.1%  Residues in additional allowed regions: 16.8%  Residues in generously allowed regions: 3.1%  Residues in disallowed regions: 4.1% |
|  | Model 5 | Z-Score: -0.71  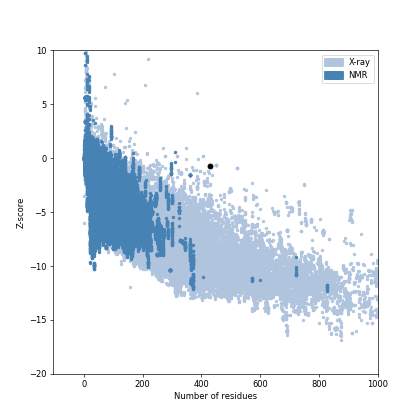 | 80 | Residues in most favored regions: 58.5%  Residues in additional allowed regions: 29.0%  Residues in generously allowed regions: 11.2%  Residues in disallowed regions: 2.3% |
| GalaxyTMB | Model 1 | Z-Score: -1.49  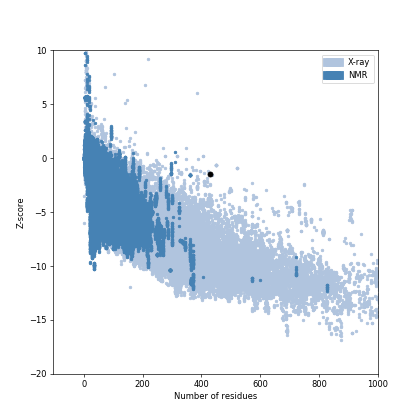 | 81.6327 | Residues in most favored regions: 85.0%  Residues in additional allowed regions: 12.2%  Residues in generously allowed regions: 1.5%  Residues in disallowed regions: 1.3% |
|  | Model 2 | Z-Score: -1.33  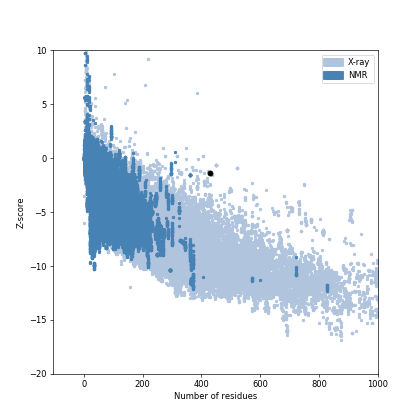 | 83.1633 | Residues in most favored regions: 85.8%  Residues in additional allowed regions: 10.9%  Residues in generously allowed regions: 1.5%  Residues in disallowed regions: 1.8% |
|  | Model 3 | Z-Score: -1.43  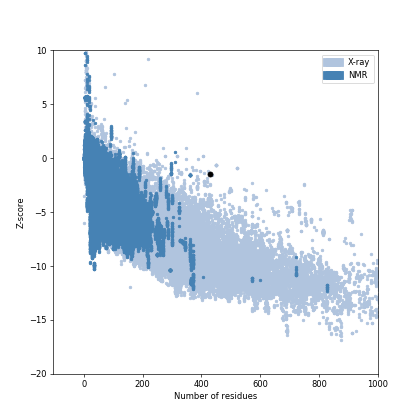 | 79.3451 | Residues in most favored regions: 85.5%  Residues in additional allowed regions: 11.7%  Residues in generously allowed regions: 1.8%  Residues in disallowed regions: 1.0% |
|  | Model 4 | Z-Score: -1.09  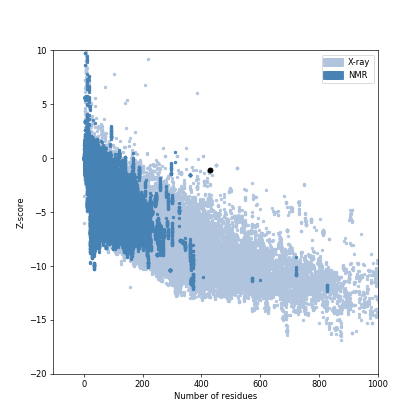 | 77.0202 | Residues in most favored regions: 84.7%  Residues in additional allowed regions: 12.5%  Residues in generously allowed regions: 1.3%  Residues in disallowed regions: 1.5% |
|  | Model 5 | Z-Score: -1.08  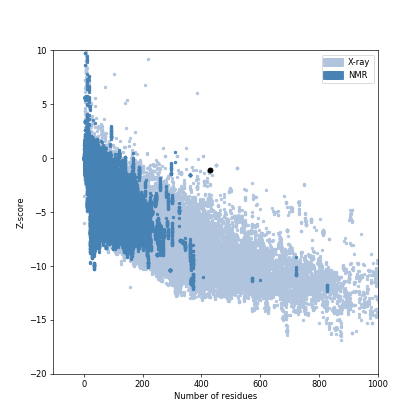 | 82.0707 | Residues in most favored regions: 85.5%  Residues in additional allowed regions: 11.2%  Residues in generously allowed regions: 1.3%  Residues in disallowed regions: 2.0% |
| trRosetta |  | Z-Score: -3.36  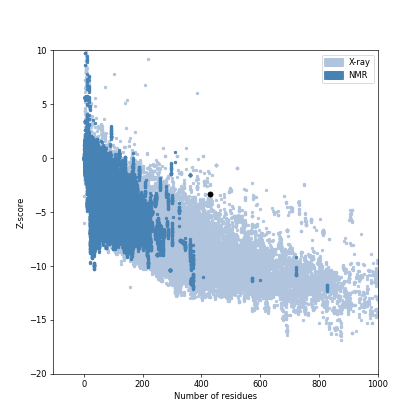 | 96.8974 | Residues in most favored regions: 90.8%  Residues in additional allowed regions: 7.6%  Residues in generously allowed regions: 0.5%  Residues in disallowed regions: 1.0% |
| Sequence 2 | | | | |
| I-TASSER | Model 1 | Z-Score: -4.79  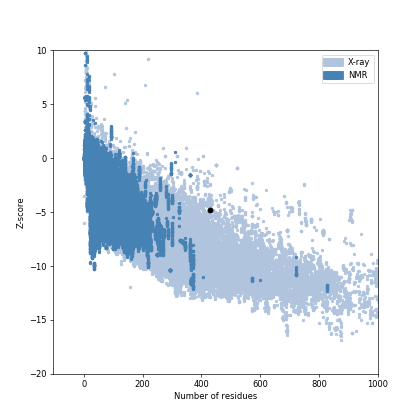 | 88.8889 | Residues in most favored regions: 85.3%  Residues in additional allowed regions: 11.4%  Residues in generously allowed regions: 2.3%  Residues in disallowed regions: 1.0% |
|  | Model 2 | Z-Score: -4.69  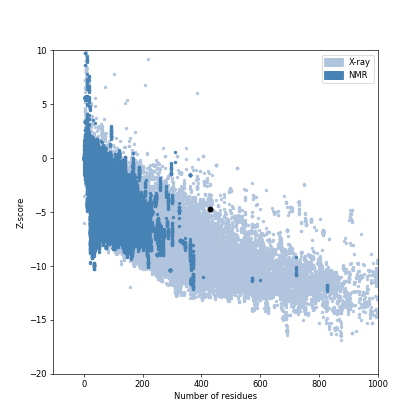 | 97.381 | Residues in most favored regions: 81.0%  Residues in additional allowed regions: 14.4%  Residues in generously allowed regions: 2.3%  Residues in disallowed regions: 2.3% |
|  | Model 3 | Z-Score: -6.05  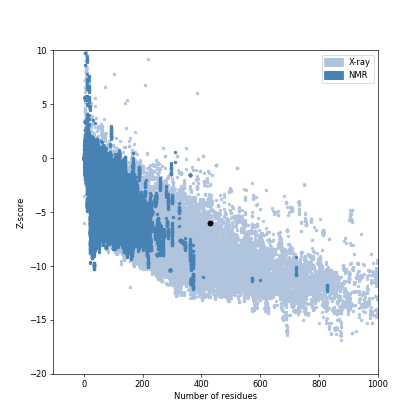 | 94.5238 | Residues in most favored regions: 81.5%  Residues in additional allowed regions: 14.4%  Residues in generously allowed regions: 2.5%  Residues in disallowed regions: 1.5% |
|  | Model 4 | Z-Score: -2.94  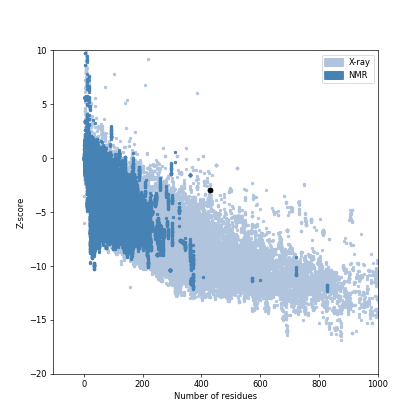 | 84.058 | Residues in most favored regions: 57.5%  Residues in additional allowed regions: 32.2%  Residues in generously allowed regions: 7.6%  Residues in disallowed regions: 2.8% |
|  | Model 5 | Z-Score: -0.64  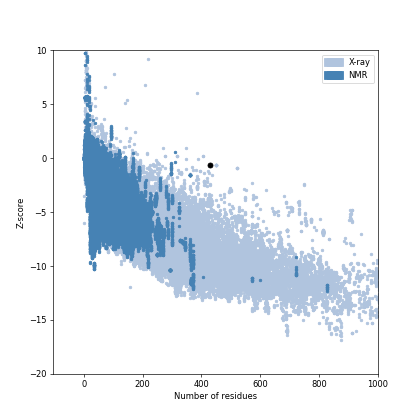 | 69.5332 | Residues in most favored regions: 54.7%  Residues in additional allowed regions: 29.6%  Residues in generously allowed regions: 11.6%  Residues in disallowed regions: 4.1% |
| GalaxyTMB | Model 1 | Z-Score: -1.91  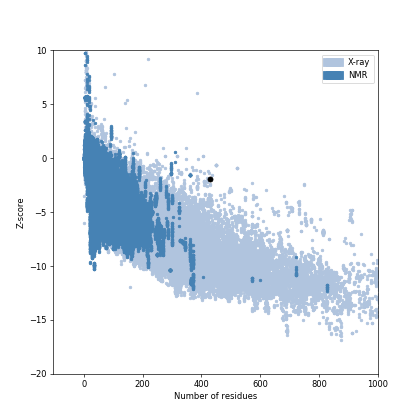 | 86.1461 | Residues in most favored regions: 86.1%  Residues in additional allowed regions: 11.4%  Residues in generously allowed regions: 1.5%  Residues in disallowed regions: 1.0% |
|  | Model 2 | Z-Score: -1.62  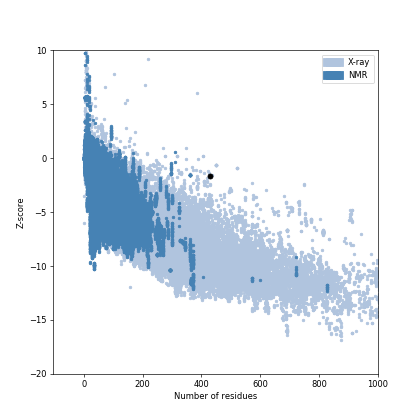 | 89.8219 | Residues in most favored regions: 85.8%  Residues in additional allowed regions: 12.4%  Residues in generously allowed regions: 0.8%  Residues in disallowed regions: 1.0% |
|  | Model 3 | Z-Score: -1.73  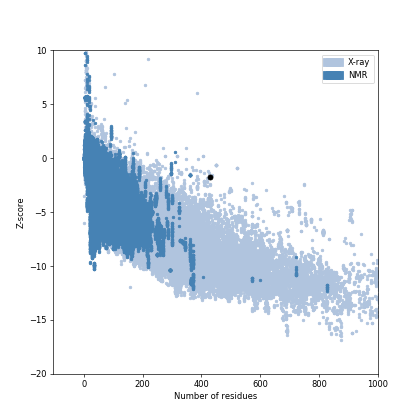 | 85.1385 | Residues in most favored regions: 86.3%  Residues in additional allowed regions: 11.6%  Residues in generously allowed regions: 1.3%  Residues in disallowed regions: 0.8% |
|  | Model 4 | Z-Score: -1.94  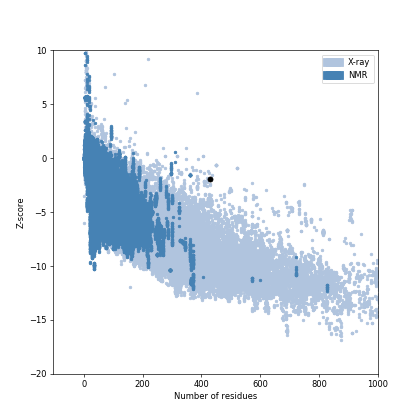 | 86.9898 | Residues in most favored regions: 85.1%  Residues in additional allowed regions: 13.2%  Residues in generously allowed regions: 1.0%  Residues in disallowed regions: 0.8% |
|  | Model 5 | Z-Score: -1.65  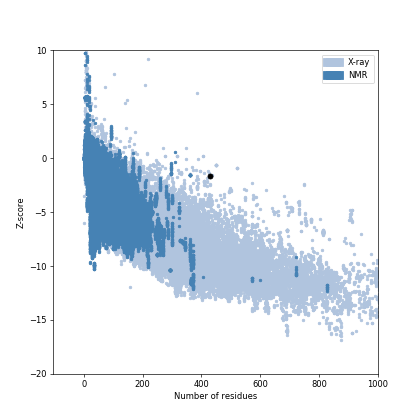 | 85.1562 | Residues in most favored regions: 84.8%  Residues in additional allowed regions: 13.7%  Residues in generously allowed regions: 1.3%  Residues in disallowed regions: 0.3% |
| Rosetta |  | Z-Score: -2.07  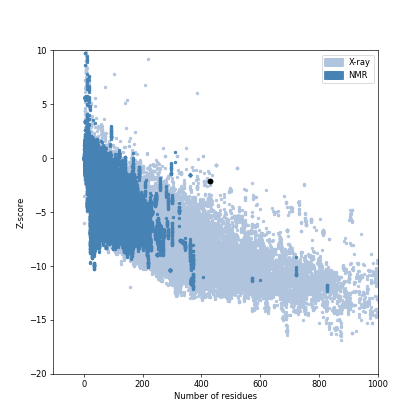 | 97.3171 | Residues in most favored regions: 93.9%  Residues in additional allowed regions: 5.1%  Residues in generously allowed regions: 0.0%  Residues in disallowed regions: 1.0% |
| Sequence 3 | | | | |
| I-TASSER | Model 1 | Z-Score: -4.65  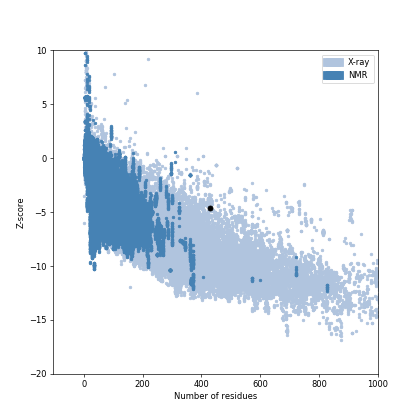 | 95 | Residues in most favored regions: 79.6%  Residues in additional allowed regions: 17.0%  Residues in generously allowed regions: 1.8%  Residues in disallowed regions: 1.5% |
|  | Model 2 | Z-Score: -3.88  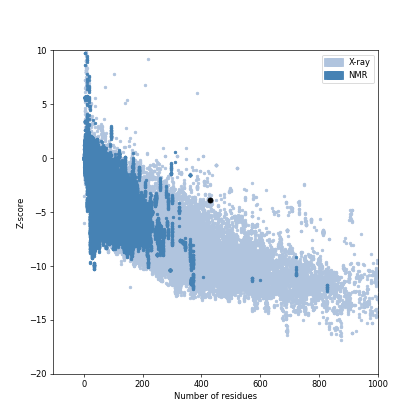 | 83.0952 | Residues in most favored regions: 76.1%  Residues in additional allowed regions: 16.1%  Residues in generously allowed regions: 5.3%  Residues in disallowed regions: 2.5% |
|  | Model 3 | Z-Score: -2.03  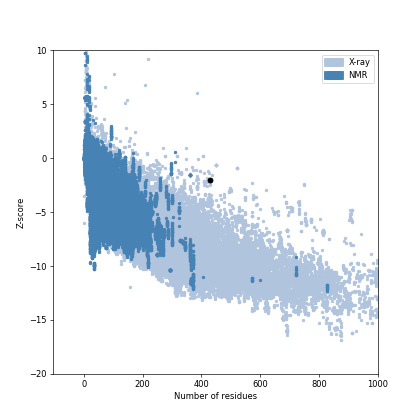 | 79.9523 | Residues in most favored regions: 66.9%  Residues in additional allowed regions: 24.9%  Residues in generously allowed regions: 5.6%  Residues in disallowed regions: 2.5% |
|  | Model 4 | Z-Score: -1.86  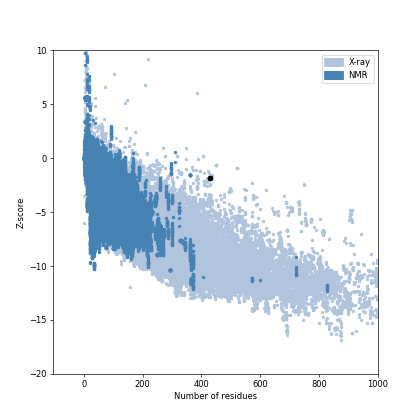 | 75 | Residues in most favored regions: 53.9%  Residues in additional allowed regions: 32.8%  Residues in generously allowed regions: 10.2%  Residues in disallowed regions: 3.1% |
|  | Model 5 | Z-Score: -2.12  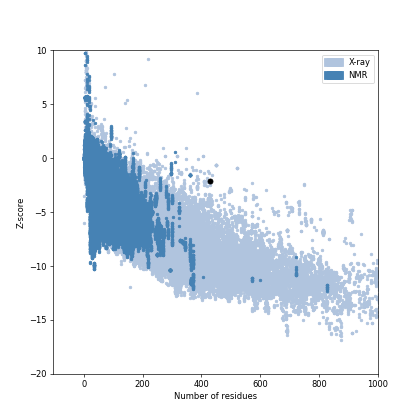 | 70.7143 | Residues in most favored regions: 57.8%  Residues in additional allowed regions: 35.9%  Residues in generously allowed regions: 4.8%  Residues in disallowed regions: 1.5% |
| GalaxyTMB | Model 1 | Z-Score: -1.9  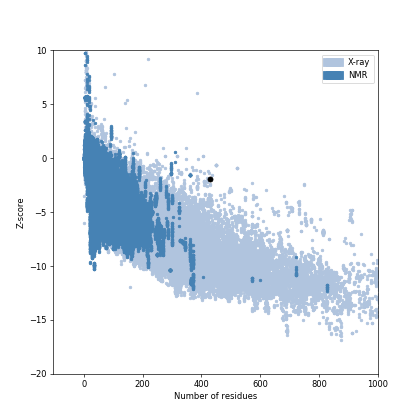 | 87.7805 | Residues in most favored regions: 86.0%  Residues in additional allowed regions: 13.0%  Residues in generously allowed regions: 0.8%  Residues in disallowed regions: 0.3% |
|  | Model 2 | Z-Score: -2.15  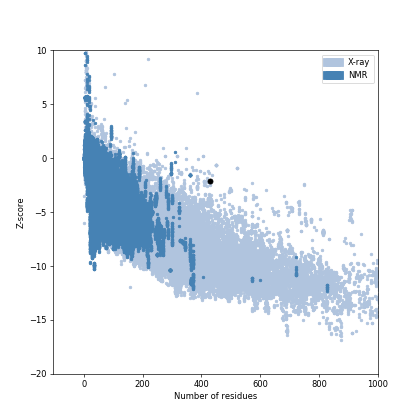 | 87.3134 | Residues in most favored regions: 87.0%  Residues in additional allowed regions: 10.3%  Residues in generously allowed regions: 1.3%  Residues in disallowed regions: 1.0% |
|  | Model 3 | Z-Score: -1.96  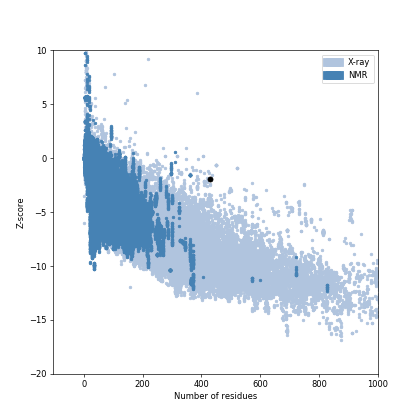 | 89.6296 | Residues in most favored regions: 87.0%  Residues in additional allowed regions: 11.5%  Residues in generously allowed regions: 0.8%  Residues in disallowed regions: 0.8% |
|  | Model 4 | Z-Score: -1.99  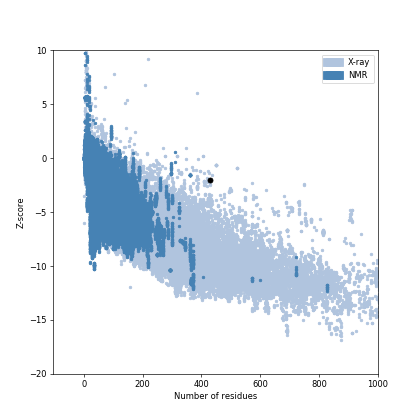 | 91.25 | Residues in most favored regions: 89.3%  Residues in additional allowed regions: 9.2%  Residues in generously allowed regions: 0.5%  Residues in disallowed regions: 1.0% |
|  | Model 5 | Z-Score: -2.04  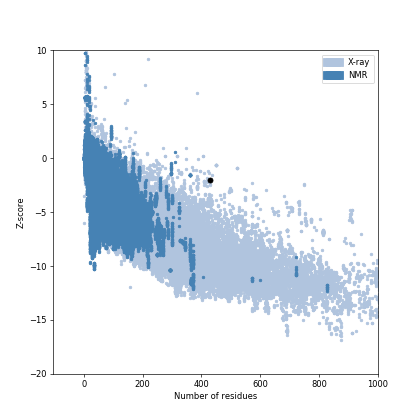 | 91.067 | Residues in most favored regions: 87.0%  Residues in additional allowed regions: 11.7%  Residues in generously allowed regions: 0.5%  Residues in disallowed regions: 0.8% |
| Rosetta |  | Z-Score: -3.76  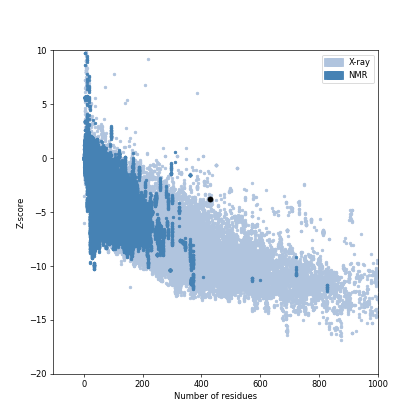 | 98.3294 | Residues in most favored regions: 93.6%  Residues in additional allowed regions: 5.1%  Residues in generously allowed regions: 0.8%  Residues in disallowed regions: 0.5% |
| Sequence 4 | | | | |
| I-TASSER | Model 1 | Z-Score: -4.23  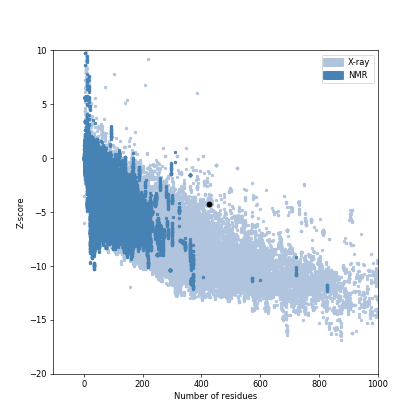 | 93.9173 | Residues in most favored regions: 77.7%  Residues in additional allowed regions: 15.5%  Residues in generously allowed regions: 4.6%  Residues in disallowed regions: 2.3% |
|  | Model 2 | Z-Score: -3.53  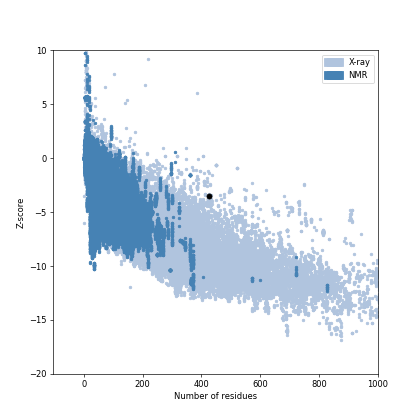 | 96.42 | Residues in most favored regions: 85.8%  Residues in additional allowed regions: 12.2%  Residues in generously allowed regions: 0.3%  Residues in disallowed regions: 1.8% |
|  | Model 3 | Z-Score: -4.6  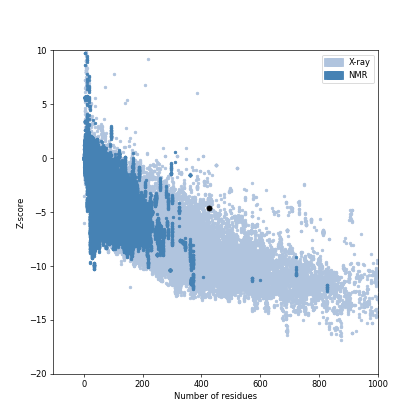 | 89.2601 | Residues in most favored regions: 77.4%  Residues in additional allowed regions: 17.0%  Residues in generously allowed regions: 3.6%  Residues in disallowed regions: 2.0% |
|  | Model 4 | Z-Score: -4.56  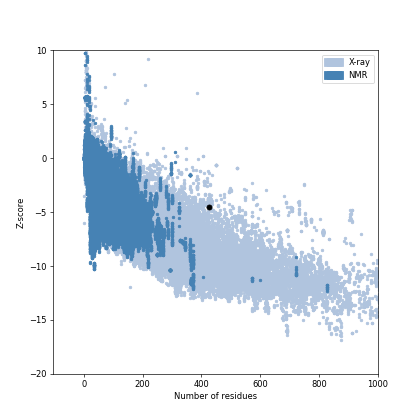 | 82.5776 | Residues in most favored regions: 74.9%  Residues in additional allowed regions: 16.0%  Residues in generously allowed regions: 4.8%  Residues in disallowed regions: 4.3% |
|  | Model 5 | Z-Score: -1.03  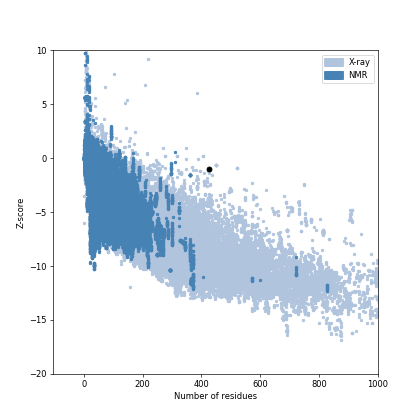 | 71.8447 | Residues in most favored regions: 59.9%  Residues in additional allowed regions: 30.7%  Residues in generously allowed regions: 5.6%  Residues in disallowed regions: 3.8% |
| GalaxyTMB | Model 1 | Z-Score: -0.93  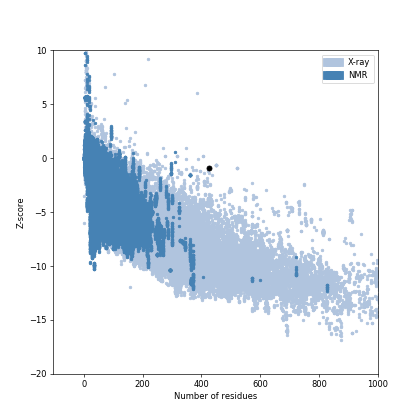 | 71.466 | Residues in most favored regions: 81.7%  Residues in additional allowed regions: 16.2%  Residues in generously allowed regions: 1.3%  Residues in disallowed regions: 0.8% |
|  | Model 2 | Z-Score: -1.05  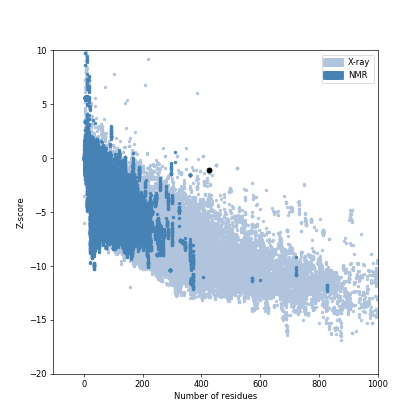 | 84.4327 | Residues in most favored regions: 83.0%  Residues in additional allowed regions: 14.7%  Residues in generously allowed regions: 1.3%  Residues in disallowed regions: 1.0% |
|  | Model 3 | Z-Score: -1.09  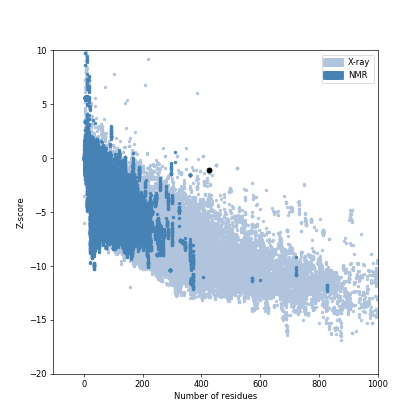 | 79.5213 | Residues in most favored regions: 82.5%  Residues in additional allowed regions: 15.5%  Residues in generously allowed regions: 1.8%  Residues in disallowed regions: 0.3% |
|  | Model 4 | Z-Score: -1.15  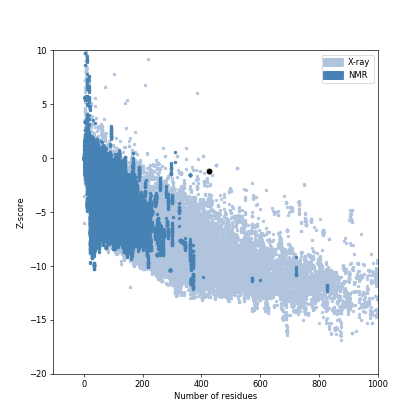 | 76.178 | Residues in most favored regions: 81.2%  Residues in additional allowed regions: 16.0%  Residues in generously allowed regions: 1.8%  Residues in disallowed regions: 1.0% |
|  | Model 5 | Z-Score: -1.16  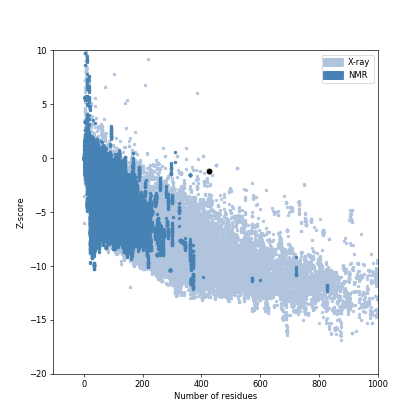 | 71.2794 | Residues in most favored regions: 80.5%  Residues in additional allowed regions: 17.5%  Residues in generously allowed regions: 1.0%  Residues in disallowed regions: 1.0% |
| Rosetta |  | Z-Score: -3.65  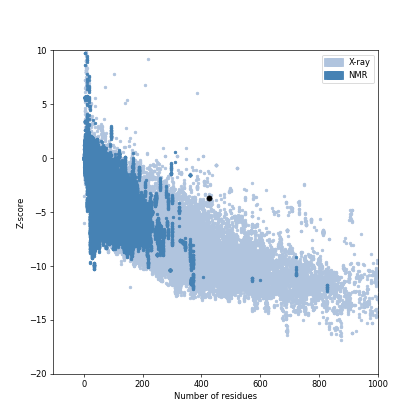 | 90.8213 | Residues in most favored regions: 89.6%  Residues in additional allowed regions: 7.1%  Residues in generously allowed regions: 1.8%  Residues in disallowed regions: 1.5% |
| Sequence 5 | | | | |
| I-TASSER | Model 1 | Z-Score: -6.34  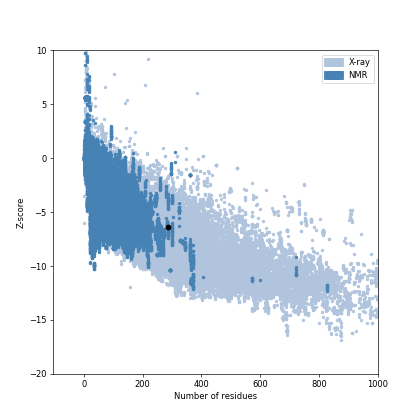 | 96.0289 | Residues in most favored regions: 82.2%  Residues in additional allowed regions: 14.3%  Residues in generously allowed regions: 1.9%  Residues in disallowed regions: 1.5% |
|  | Model 2 | Z-Score: -4.52  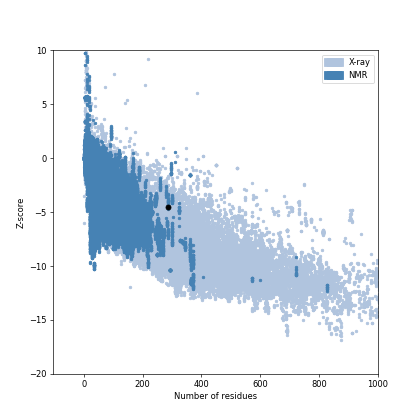 | 89.5307 | Residues in most favored regions: 70.7%  Residues in additional allowed regions: 22.4%  Residues in generously allowed regions: 3.5%  Residues in disallowed regions: 3.5% |
|  | Model 3 | Z-Score: -5.03  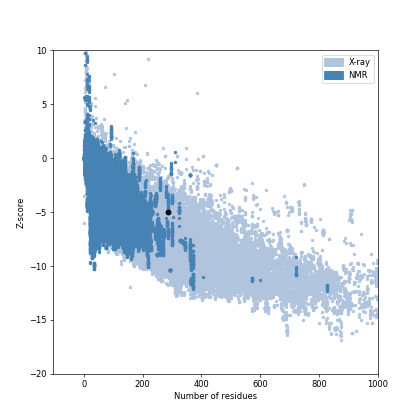 | 92.7798 | Residues in most favored regions: 69.1%  Residues in additional allowed regions: 26.6%  Residues in generously allowed regions: 1.2%  Residues in disallowed regions: 3.1% |
|  | Model 4 | Z-Score: -5.06  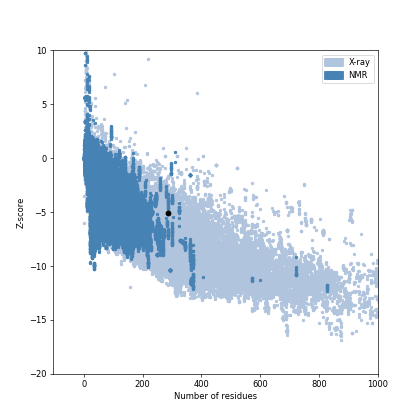 | 94.2238 | Residues in most favored regions: 69.1%  Residues in additional allowed regions: 25.1%  Residues in generously allowed regions: 2.7%  Residues in disallowed regions: 3.1% |
|  | Model 5 | Z-Score: -4.47  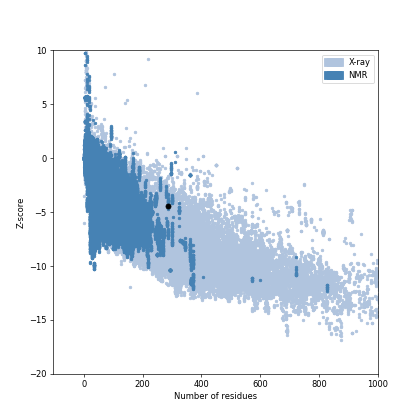 | 76.5343 | Residues in most favored regions: 67.2%  Residues in additional allowed regions: 25.9%  Residues in generously allowed regions: 3.1%  Residues in disallowed regions: 3.9% |
| GalaxyTMB | Model 1 | Z-Score: -0.74  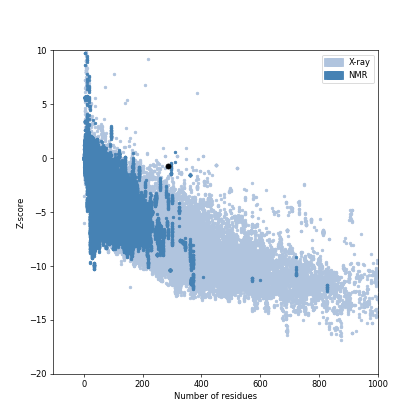 | 76.4228 | Residues in most favored regions: 85.3%  Residues in additional allowed regions: 13.9%  Residues in generously allowed regions: 0.4%  Residues in disallowed regions: 0.4% |
|  | Model 2 | Z-Score: -0.57  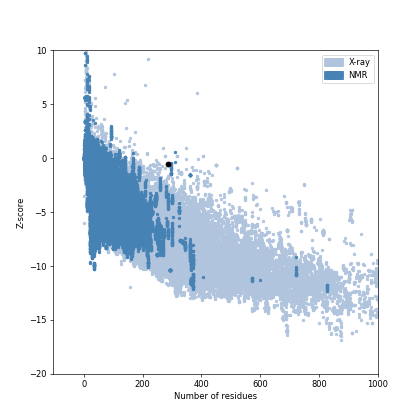 | 79.4574 | Residues in most favored regions: 86.5%  Residues in additional allowed regions: 12.4%  Residues in generously allowed regions: 0.4%  Residues in disallowed regions: 0.8% |
|  | Model 3 | Z-Score: -0.66  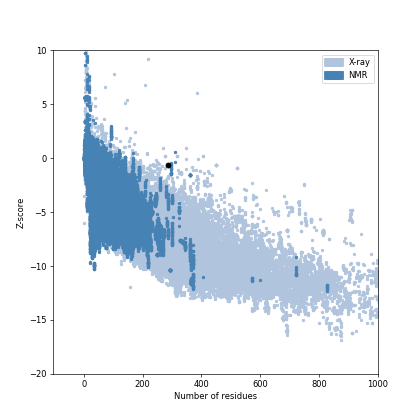 | 75.7202 | Residues in most favored regions: 86.9%  Residues in additional allowed regions: 12.0%  Residues in generously allowed regions: 0.4%  Residues in disallowed regions: 0.8% |
|  | Model 4 | Z-Score: -0.6  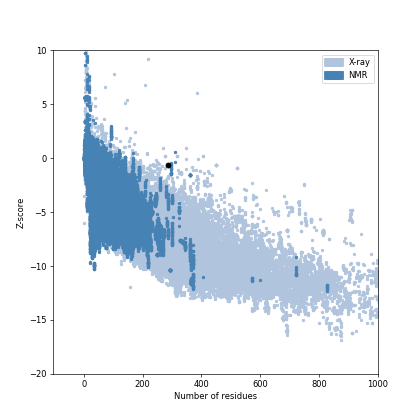 | 75.6 | Residues in most favored regions: 87.3%  Residues in additional allowed regions: 12.0%  Residues in generously allowed regions: 0.4%  Residues in disallowed regions: 0.4% |
|  | Model 5 | Z-Score: -0.59  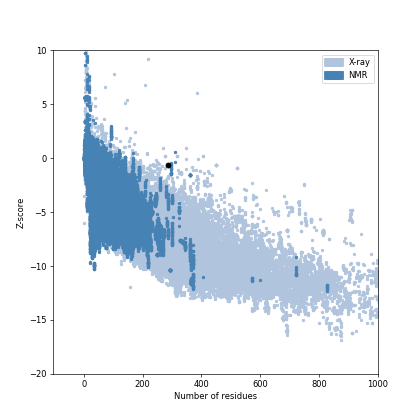 | 77.9116 | Residues in most favored regions: 86.5%  Residues in additional allowed regions: 12.4%  Residues in generously allowed regions: 0.8%  Residues in disallowed regions: 0.4% |
| Rosetta |  | Z-Score: -7.29  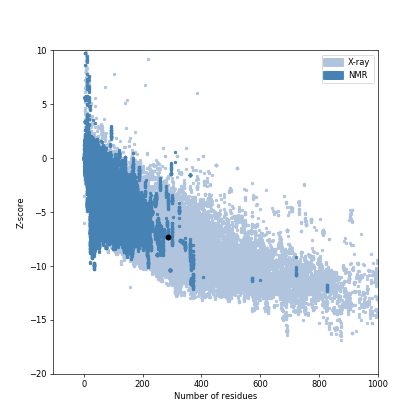 | 91.3357 | Residues in most favored regions: 87.3%  Residues in additional allowed regions: 10.8%  Residues in generously allowed regions: 1.2%  Residues in disallowed regions: 0.8% |
| Sequence 6 | | | | |
| I-TASSER | Model 1 | Z-Score: -2.49  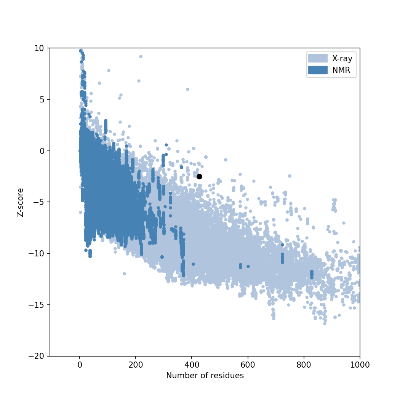 | 90.5109 | Residues in most favored regions: 66.0%  Residues in additional allowed regions: 28.2%  Residues in generously allowed regions: 3.6%  Residues in disallowed regions: 2.3% |
|  | Model 2 | Z-Score: -1.94  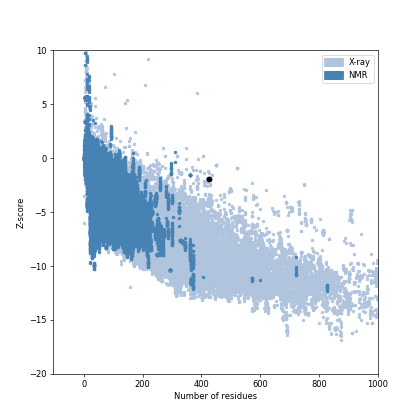 | 72.9665 | Residues in most favored regions: 58.1%  Residues in additional allowed regions: 28.4%  Residues in generously allowed regions: 8.6%  Residues in disallowed regions: 4.8% |
|  | Model 3 | Z-Score: -3.13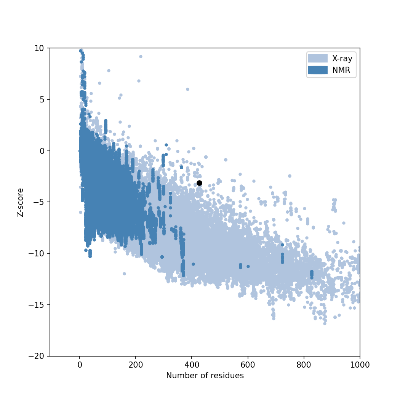 | 73.8499 | Residues in most favored regions: 59.1%  Residues in additional allowed regions: 27.9%  Residues in generously allowed regions: 8.4%  Residues in disallowed regions: 4.6% |
|  | Model 4 | Z-Score: -4.03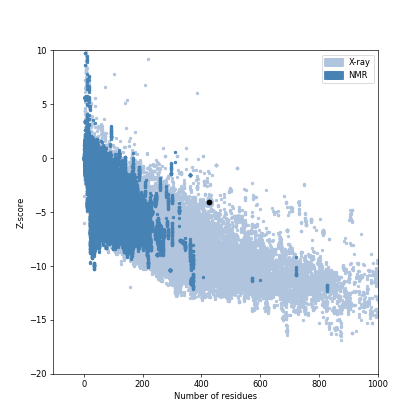 | 85.9189 | Residues in most favored regions: 75.1%  Residues in additional allowed regions: 17.0%  Residues in generously allowed regions: 5.3%  Residues in disallowed regions: 2.5% |
|  | Model 5 | Z-Score: -3.33  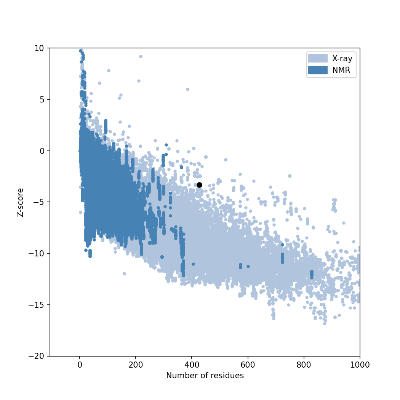 | 94.2446 | Residues in most favored regions: 66.2%  Residues in additional allowed regions: 25.1%  Residues in generously allowed regions: 4.8%  Residues in disallowed regions: 3.8% |
| GalaxyTMB | Model 1 | Z-Score: 0.08  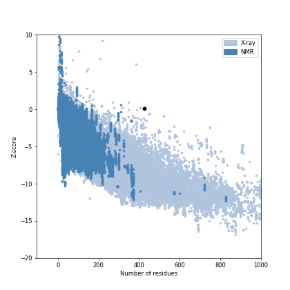 | 72.9469 | Residues in most favored regions: 89.1%  Residues in additional allowed regions: 8.9%  Residues in generously allowed regions: 1.0%  Residues in disallowed regions: 1.0% |
|  | Model 2 | Z-Score: 0.25  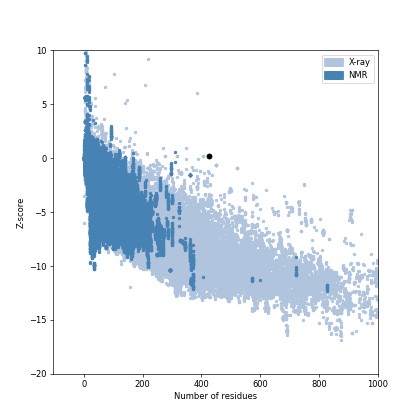 | 75.9305 | Residues in most favored regions: 91.4%  Residues in additional allowed regions: 6.9%  Residues in generously allowed regions: 0.5%  Residues in disallowed regions: 1.3% |
|  | Model 3 | Z-Score: -0.34  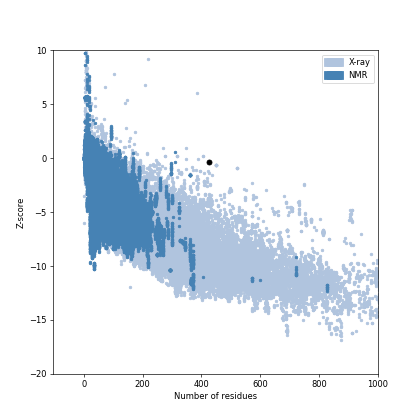 | 84.9754 | Residues in most favored regions: 90.4%  Residues in additional allowed regions: 8.4%  Residues in generously allowed regions: 0.8%  Residues in disallowed regions: 0.5% |
|  | Model 4 | Z-Score: 0.36  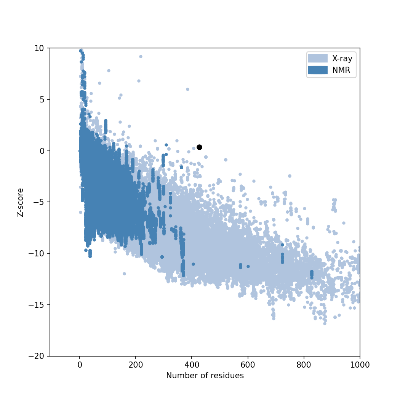 | 79.602 | Residues in most favored regions: 90.9%  Residues in additional allowed regions: 7.6%  Residues in generously allowed regions: 0.5%  Residues in disallowed regions: 1.0% |
|  | Model 5 | Z-Score: 0.25  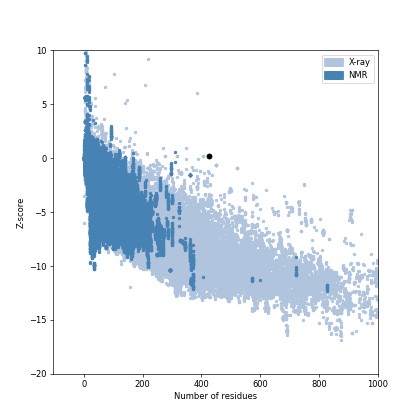 | 70.25 | Residues in most favored regions: 90.1%  Residues in additional allowed regions: 8.4%  Residues in generously allowed regions: 1.0%  Residues in disallowed regions: 0.5% |
| Rosetta |  | Z-Score: -3.84  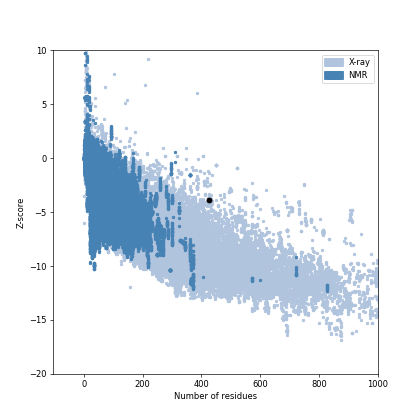 | 91.3253 | Residues in most favored regions: 90.9%  Residues in additional allowed regions: 8.4%  Residues in generously allowed regions: 0.5%  Residues in disallowed regions: 0.3% |
