# Supplementary material for: Design and in silico evaluation of an mRNA vaccine against HTLV-1 using AI-driven reverse vaccinology approaches
Source: PLoS One. 2026 May 6;21(5):e0340201. doi: 10.1371/journal.pone.0340201 (PMC13148667; doi:10.1371/journal.pone.0340201)
Supplement: S4 Table — (DOCX) [file pone.0340201.s005.docx]

**S4 Table.** Evaluation results of refined 3D structures from the selected structure in the previous step.

| Models | Prosa web | ERRAT (Overall Quality Factor) | Ramachandran plot |
| --- | --- | --- | --- |
| Sequence 5- Rosetta 3D structure | | | |
| Model 1 | Z-Score: -7.59  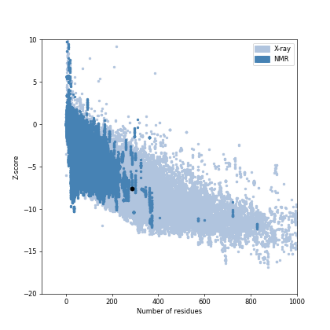 | 95.6679 | Residues in most favored regions: 92.3%  Residues in additional allowed regions: 5.8%  Residues in generously allowed regions: 1.2%  Residues in disallowed regions: 0.8% |
| Model 2 | Z-Score: -7.47  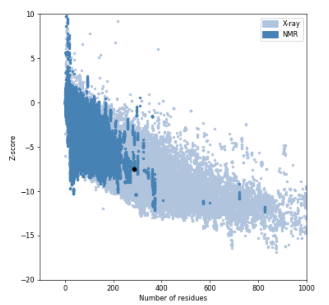 | 95.3069 | Residues in most favored regions: 91.1%  Residues in additional allowed regions: 5.8%  Residues in generously allowed regions: 1.5%  Residues in disallowed regions: 1.5% |
| Model 3 | Z-Score: -7.55  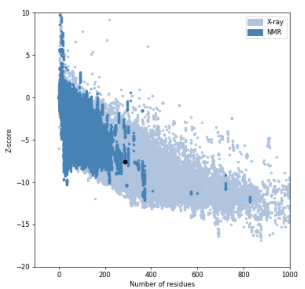 | 95.6679 | Residues in most favored regions: 90.7%  Residues in additional allowed regions: 7.3%  Residues in generously allowed regions: 1.2%  Residues in disallowed regions: 0.8% |
| Model 4 | Z-Score: -7.47  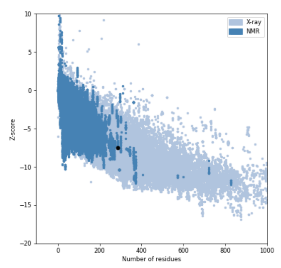 | 95.3069 | Residues in most favored regions: 91.9%  Residues in additional allowed regions: 6.2%  Residues in generously allowed regions: 1.2%  Residues in disallowed regions: 0.8% |
| Model 5 | Z-Score: -7.64  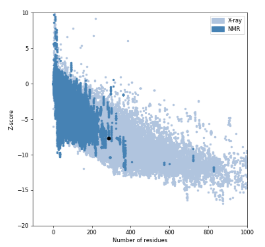 | 95.3069 | Residues in most favored regions: 91.9%  Residues in additional allowed regions: 5.4%  Residues in generously allowed regions: 1.5%  Residues in disallowed regions: 1.2% |
| Model 6 | Z-Score: -7.48  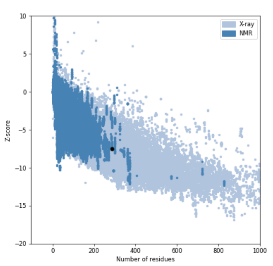 | 93.5018 | Residues in most favored regions: 91.9%  Residues in additional allowed regions: 5.4%  Residues in generously allowed regions: 1.5%  Residues in disallowed regions: 1.2% |
| Model 7 | Z-Score: -7.35  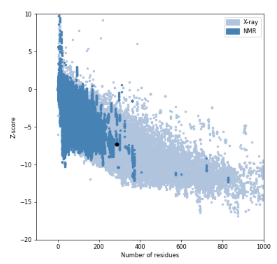 | 93.8628 | Residues in most favored regions: 89.6%  Residues in additional allowed regions: 7.7%  Residues in generously allowed regions: 1.9%  Residues in disallowed regions: 0.8% |
| Model 8 | Z-Score: -7.65  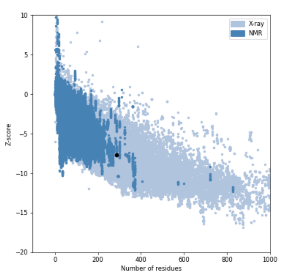 | 94.2238 | Residues in most favored regions: 91.5%  Residues in additional allowed regions: 5.8%  Residues in generously allowed regions: 1.5%  Residues in disallowed regions: 1.2% |
| Model 9 | Z-Score: -7.54  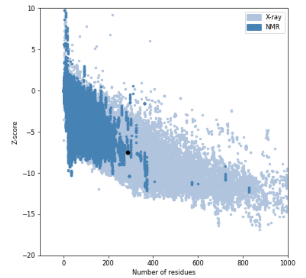 | 92.7798 | Residues in most favored regions: 91.5%  Residues in additional allowed regions: 5.8%  Residues in generously allowed regions: 1.5%  Residues in disallowed regions: 1.2% |
| Model 10 | Z-Score: -7.53  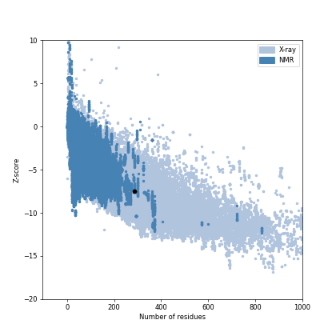 | 98.556 | Residues in most favored regions: 91.5%  Residues in additional allowed regions: 5.8%  Residues in generously allowed regions: 1.5%  Residues in disallowed regions: 1.2% |
