# Supplementary material for: Design and in silico evaluation of an mRNA vaccine against HTLV-1 using AI-driven reverse vaccinology approaches
Source: PLoS One. 2026 May 6;21(5):e0340201. doi: 10.1371/journal.pone.0340201 (PMC13148667; doi:10.1371/journal.pone.0340201)
Supplement: S5 Table — (DOCX) [file pone.0340201.s006.docx]

**S5 Table.** The impact of mutations of HIS 105 and VAL 207 on the vaccine’s stability

| **Amino acid** | **Overall Stability** | **Torsion*** | **Predicted ΔΔG (kcal/mol)** | |
| --- | --- | --- | --- | --- |
| **HIS 105** | | | | |
| GLY | Stabilising | Favourable | 0.36 | |
| ALA | Destabilising | Unfavourable | -0.09 | |
| VAL | Destabilising | Unfavourable | -1.12 | |
| LEU | Stabilising | Unfavourable | 0.42 | |
| ILE | Stabilising | Unfavourable | 0.43 | |
| MET | Stabilising | Unfavourable | 0.12 | |
| PRO | Destabilising | Unfavourable | -0.17 | |
| TRP | Destabilising | Unfavourable | -1.91 | |
| SER | Stabilising | Favourable | 0.1 | |
| THR | Stabilising | Favourable | 0.16 | |
| PHE | Destabilising | Unfavourable | -1.12 | |
| GLN | Destabilising | Favourable | -0.19 | |
| LYS | Stabilising | Favourable | 0.28 | |
| TYR | Destabilising | Favourable | -0.09 | |
| ASN | Stabilising | Favourable | 0.18 | |
| CYS | Stabilising | Unfavourable | 0.13 | |
| GLU | Stabilising | Favourable | 0.4 | |
| ASP | Stabilising | Favourable | 0.07 | |
| ARG | Destabilising | Unfavourable | -0.58 | |
| **VAL 207** | | | | |
| GLY | Destabilising | Favourable | | -1.19 |
| ALA | Destabilising | Unfavourable | | -1.38 |
| LEU | Destabilising | Unfavourable | | -0.81 |
| ILE | Destabilising | Favourable | | -0.33 |
| MET | Destabilising | Favourable | | -3.13 |
| PRO | Destabilising | Unfavourable | | -1.48 |
| TRP | Destabilising | Favourable | | -0.18 |
| SER | Destabilising | Favourable | | -0.65 |
| THR | Destabilising | Unfavourable | | -0.63 |
| PHE | Destabilising | Unfavourable | | -1.05 |
| GLN | Destabilising | Unfavourable | | -0.06 |
| LYS | Destabilising | Unfavourable | | -1.72 |
| TYR | Destabilising | Unfavourable | | -0.4 |
| ASN | Destabilising | Unfavourable | | -0.8 |
| CYS | Destabilising | Favourable | | -2.47 |
| GLU | Destabilising | Favourable | | -0.61 |
| ASP | Destabilising | Favourable | | -1.92 |
| ARG | Destabilising | Unfavourable | | -0.98 |
| HIS | Destabilising | Favourable | | -0.27 |
