# Supplementary material for: Design and in silico evaluation of an mRNA vaccine against HTLV-1 using AI-driven reverse vaccinology approaches
Source: PLoS One. 2026 May 6;21(5):e0340201. doi: 10.1371/journal.pone.0340201 (PMC13148667; doi:10.1371/journal.pone.0340201)
Supplement: S6 Table — (DOCX) [file pone.0340201.s007.docx]

**Table S6**. URL and validity of the used servers in this study

| Server | | Accuracy | Sensitivity | Specificity | TM-score | Positive predictive value | Correlation coefficients |
| --- | --- | --- | --- | --- | --- | --- | --- |
| UniProt database (UniProtKB) | |  |  |  |  |  |  |
| ANTIGENpro | | 76% [1] |  |  |  |  |  |
| VaxiJen v2.0 | | 70 to 89% [2] |  |  |  |  |  |
| AllerTOP v2.0 | | 88.7% [3] | 94% [3] | 94% [3] |  |  |  |
| AllergenFP v.1.0 | | 0.879 [4] | 0.868 [4] | 0.891 [4] |  |  |  |
| CTLpred | | more than 72% [5] |  |  |  |  |  |
| IEDB | |  |  |  |  |  |  |
| Rankpep | |  |  |  |  |  |  |
| BCPREDS | |  |  |  |  |  |  |
| IEDB (BepiPred-2.0) | | 51.93 [6] | 63.35 [6] | 42.63 [6] |  |  |  |
| ToxinPred2 | | 95.54 [7] | 93.69 [7] | 97.39 [7] |  |  |  |
| I-TASSER | |  |  |  | 0.43±0.14 |  |  |
| GalaxyTBM | |  |  |  |  |  |  |
| trRosetta | |  |  |  | 0.404 |  |  |
| ProSA-web | |  |  |  |  |  |  |
| SAVESv6.0 | |  |  |  |  |  |  |
| GalaxyLoop | |  |  |  |  |  |  |
| GalaxyRefine2 | |  |  |  |  |  |  |
| SCooP | |  |  |  |  |  |  |
| ProtParam | |  |  |  |  |  |  |
| ElliPro | | 80% to 90% [8] |  |  |  |  |  |
| ClusPro | | ˜70% [9] |  |  |  |  |  |
| PDBsum | |  |  |  |  |  |  |
| PPCheck | | 72.18 [10] | 58.798 [10] | 77.928 [10] |  |  |  |
| PRODIGY | |  |  |  |  |  |  |
| GenSmart Codon Optimization | |  |  |  |  |  |  |
| DNA>RNA>Protein | |  |  |  |  |  |  |
| RNAfold (ViennaRNA Package 2.0) | |  | 0.742 [11] |  |  | 0.795 [11] |  |
| NetNGlyc 1.0 | | 93% [12] | 75% [12] |  |  |  |  |
| NetPhos 3.1 | serine | 81.13% [13] | 16.59% [13] | 81.91% [13] |  |  |  |
|  | threonine | 82.19% [13] | 22.35% [13] | 82.66% [13] |  |  |  |
|  | tyrosine | 82.96% [13] | 16.49% [13] | 84.16% [13] |  |  |  |
| NetAcet 1.0 | |  | up to 74% [14] |  |  |  | close to 0.7 [14] |
| big-PI/GPI animals | | 83.3% [15] |  |  |  |  |  |
| MyrPS/NMT | |  |  |  |  |  |  |
| Population coverage prediction tool of IEDB | |  |  |  |  |  |  |
| C-ImmSim | |  |  |  |  |  |  |

**References:**

1. Magnan, C.N., et al., *High-throughput prediction of protein antigenicity using protein microarray data.* Bioinformatics, 2010. **26**(23): p. 2936-2943.

2. Doytchinova, I.A. and D.R. Flower, *VaxiJen: a server for prediction of protective antigens, tumour antigens and subunit vaccines.* BMC bioinformatics, 2007. **8**: p. 1-7.

3. Dimitrov, I., et al., *AllerTOP v. 2—a server for in silico prediction of allergens.* Journal of molecular modeling, 2014. **20**: p. 1-6.

4. Dimitrov, I., et al., *AllergenFP: allergenicity prediction by descriptor fingerprints.* Bioinformatics, 2014. **30**(6): p. 846-851.

5. Bhasin, M. and G.P. Raghava, *Prediction of CTL epitopes using QM, SVM and ANN techniques.* Vaccine, 2004. **22**(23-24): p. 3195-3204.

6. Galanis, K.A., et al., *Linear B-cell epitope prediction for in silico vaccine design: A performance review of methods available via command-line interface.* International journal of molecular sciences, 2021. **22**(6): p. 3210.

7. Sharma, N., et al., *ToxinPred2: an improved method for predicting toxicity of proteins.* Briefings in bioinformatics, 2022. **23**(5): p. bbac174.

8. Ponomarenko, J., et al., *ElliPro: a new structure-based tool for the prediction of antibody epitopes.* BMC bioinformatics, 2008. **9**: p. 1-8.

9. Kozakov, D., et al., *The ClusPro web server for protein–protein docking.* Nature protocols, 2017. **12**(2): p. 255-278.

10. Sukhwal, A. and R. Sowdhamini, *PPCheck: A webserver for the quantitative analysis of protein-protein interfaces and prediction of residue hotspots.* Bioinformatics and biology insights, 2015. **9**: p. BBI. S25928.

11. Al Tbeishat, H., *Novel In Silico mRNA vaccine design exploiting proteins of M. tuberculosis that modulates host immune responses by inducing epigenetic modifications.* Scientific Reports, 2022. **12**(1): p. 4645.

12. Taherzadeh, G., et al., *SPRINT-Gly: predicting N-and O-linked glycosylation sites of human and mouse proteins by using sequence and predicted structural properties.* Bioinformatics, 2019. **35**(20): p. 4140-4146.

13. Biswas, A.K., N. Noman, and A.R. Sikder, *Machine learning approach to predict protein phosphorylation sites by incorporating evolutionary information.* BMC bioinformatics, 2010. **11**: p. 1-17.

14. Kiemer, L., J.D. Bendtsen, and N. Blom, *NetAcet: prediction of N-terminal acetylation sites.* Bioinformatics, 2005. **21**(7): p. 1269-1270.

15. Eisenhaber, B., P. Bork, and F. Eisenhaber, *Prediction of potential GPI-modification sites in proprotein sequences.* Journal of molecular biology, 1999. **292**(3): p. 741-758.
