# Supplementary material for: Design and in silico evaluation of an mRNA vaccine against HTLV-1 using AI-driven reverse vaccinology approaches
Source: PLoS One. 2026 May 6;21(5):e0340201. doi: 10.1371/journal.pone.0340201 (PMC13148667; doi:10.1371/journal.pone.0340201)
Supplement: Attachment — Submitted filename: F-Response to reviewer.docx [file pone.0340201.s009.docx]

Dear Editor,

Your comments and the reviewer's insights were highly insightful and enabled us to greatly improve the quality of our manuscript. The following pages are our amendments on journal requirements and our one-by-one responses to each of the reviewers' comments.

Revisions in the text are shown using track changes and highlights. Furthermore, the clear version of the revised manuscript (without track changes) was uploaded to the e-Component section.

Journal Requirements:

When submitting your revision, we need you to address these additional requirements.

1. Please ensure that your manuscript meets PLOS ONE's style requirements, including those for file naming. The PLOS ONE style templates can be found at

https://journals.plos.org/plosone/s/file?id=wjVg/PLOSOne_formatting_sample_main_body.pdf and

<https://journals.plos.org/plosone/s/file?id=ba62/PLOSOne_formatting_sample_title_authors_affiliations.pdf>

Done accordingly.

2. Please note that PLOS ONE has specific guidelines on code sharing for submissions in which author-generated code underpins the findings in the manuscript. In these cases, we expect all author-generated code to be made available without restrictions upon publication of the work.

Please review our guidelines at https://journals.plos.org/plosone/s/materials-and-software-sharing#loc-sharing-code and ensure that your code is shared in a way that follows best practice and facilitates reproducibility and reuse.

Not applicable.

3. Thank you for stating the following financial disclosure:

“This work was supported by the Vice-Chancellor for Research, Shiraz University of Medical Sciences, Iran (Grant number: 28569).”

Please state what role the funders took in the study. If the funders had no role, please state: "The funders had no role in study design, data collection and analysis, decision to publish, or preparation of the manuscript." If this statement is not correct you must amend it as needed.

Please include this amended Role of Funder statement in your cover letter; we will change the online submission form on your behalf.

Done accordingly.

4. Please note that funding information should not appear in the Acknowledgments section or other areas of your manuscript. We will only publish funding information present in the Funding Statement section of the online submission form. Please remove any funding-related text from the manuscript.

Done accordingly.

5. We note that Figure 12 in your submission contain map images which may be copyrighted. All PLOS content is published under the Creative Commons Attribution License (CC BY 4.0), which means that the manuscript, images, and Supporting Information files will be freely available online, and any third party is permitted to access, download, copy, distribute, and use these materials in any way, even commercially, with proper attribution. For these reasons, we cannot publish previously copyrighted maps or satellite images created using proprietary data, such as Google software (Google Maps, Street View, and Earth).

The figure was deleted, and the data is now presented in a table (Table 10).

**Reviewer 1:**

1. Explain the reason behind selecting the HTLV-1 target proteins as “candidate proteins” to design the vaccine construct.

✔ Thank you very much for your insightful comments. Eight candidate proteins were considered based on their previous application in vaccine design in earlier studies. The rationale for using these eight proteins in previous studies was that they possess oncogenic, structural, or regulatory functions and play essential roles in the viral replication cycle and pathogenicity (Page 36 in the revised manuscript with track change 34 in the clean manuscript). Then, based on antigenicity and allergenicity assessments, the Pol and GP62 antigens were selected for the final epitope selection.

2. What specific factors were considered to optimize antigen presentation and enhance immune responses, such as epitope selection, adjuvant usage, or linker design in the vaccine construct?

✔ Various techniques were used to improve antigen presentation and immune responses. For antigen presentation, the use of flexible linkers between epitopes was employed. To enhance immune responses, it was done by using the most antigenic epitopes from the most antigenic proteins in vaccine design, along with the application of various adjuvants such as RS01, RS09, PADRE, and conserved HBHA. Moreover, adjuvants were used as built-in components of the vaccine construct. Some more related explanations were added in the discussion to highlight these points (pages 35).

3. Disulfide bridges play a crucial role in stabilizing the protein’s folded conformation; therefore, the authors are encouraged to include a section on “Disulfide engineering of the vaccine”.

✔ We added Disulfide engineering of our vaccine, and this section is included in the manuscript (Methods: page 10).

4. The authors are advised to assess the solubility of the proposed vaccine construct, as solubility is a critical factor influencing its stability and bioavailability.

✔ Thanks for your suggestion. Since the vaccine is not protein and will not be produced by cloning in *E. coli*, the usual solubility servers like SolPro, which predicts protein solubility upon overexpression in *E. coli*, would not be suitable. We added solubility analysis by Aggrescan3D, which anticipates the aggregative properties of protein and fits our purpose better (Methods: page 11).

5. Explain the reason in discussion why three different linkers are used to construct the vaccine.

✔ Both rigid and flexible linkers were utilized, including GPGPG, KK, AYY, and EAAAK. Flexible linkers can be used between epitopes to reduce junctional immunogenicity. They also contribute to the immunogenicity of multi-epitope vaccines. However, a very high protein flexibility might harm the stability of the 3D structure, so we used a complex of several different linkers. For linking adjuvants to other components, a rigid linker (EAAAK) was employed, which helps increase expression, stabilize the structure, and improve bioactivity.

6. The Ramachandran plot of the modelled vaccine structure shows one outlier residue (His105). Could you provide insights into its role in the vaccine structure, and whether it impacts the stability or functionality of the vaccine? Additionally, were any efforts made to refine or resolve this outlier during structure validation?

✔ The impact of substituting the mentioned amino acid (His105), as well as another amino acid in a similar context at position 207 (Valine), with 19 other amino acids was evaluated using the CUPSAT server. The effects of these substitutions on protein structural stability were summarized in tables 7 & 8, based on the calculated ΔΔG values.

7. Perform in silico cloning for maximum vaccine expression and to predict the reverse translation efficacy.

Since the vaccine construct is mRNA, not protein, the classical *in silico* cloning might not apply here, but DNA codon optimization was done by GenSmart Codon Optimization, and factors such as CAI, CFD, and GC content were calculated (page 31).

8. The discussion section also contains several statements that require relevant explanation and citations.

✔ The discussion was reviewed precisely, and every sentence needing a reference was cited. Added citations are shown in yellow highlights.

9. The manuscript mentions the use of artificial intelligence (AI) to construct the vaccine, but the described methodology appears to rely on online servers and databases. Could the authors elaborate on the specific role AI played in the vaccine design process? If AI was not directly employed, consider revising the description for clarity.

✔ AI is categorized as predictive, descriptive, prescriptive, or generative. Machine learning, a subset of AI, develops algorithms that improve with experience. Deep learning, a form of ML, uses neural networks to identify complex patterns (Gasperini, Baylor et al. 2025). A number of servers used in this paper use artificial neural network (ANN) based or ML approaches to predict the mentioned results.

* Gasperini, G., et al. (2025). "Vaccinology in the artificial intelligence era." Science Translational Medicine **17**(794): eadu3791.

We revised several related sentences as you suggested. (Discussion: page 34/ Introduction: page 5)

10. The authors should use other studies conducted in this field in this section.
<https://doi.org/10.3390/v14112504>
<https://doi.org/10.1007/s12026-023-09403-2>
<https://doi.org/10.1016/j.vacun.2024.04.003>
<https://doi.org/10.1007/s13205-024-04022-6>
<https://doi.org/10.1016/j.biologicals.2024.101782>

✔ The mentioned references were used to improve the manuscript (ref: 11, 67, 72, 73, 76).

**Reviewer 2:**

1. In the method section, authors must include the access link to the servers used in front of their names.

✔ Thank you very much for your helpful comments. The access links have been added accordingly.

2. In this work, only epitopes related to a specific set of alleles are predicted, and the authors must provide a logical reason for this.

✔ Prediction of HLA I and HLA II binding epitopes were done for three MHC class I supertypes, namely A*01:01, A*02:01, and A*03:01, and three MHC class II supertypes, including DRB1*01:01, DRB1*03:01, and DRB1*04:01. These epitopes were selected to optimize population coverage, taking into account their wide distribution and overlap across various populations (Methods: page 6).

3. The authors should perform disulfide engineering of the vaccine construct.

✔ We added Disulfide engineering of our vaccine, and this section is included in the manuscript (Methods: page 10).

4. The captions for the simulation results figures are very brief. Please provide more explanation about these figures.

✔ Captions were edited, and more explanations were added. Thank you for this helpful suggestion.

5. Please revise the English language of your manuscript

✔ The manuscript was carefully reviewed, and all grammatical and punctuation errors were corrected.

Again, we appreciate all your insightful comments. We worked hard to be responsive to them. Thank you for taking the time and energy to help us improve the paper.

Sincerely yours,

The corresponding author,

Manica Negahdaripour
