# Supplementary material for: Design and in silico evaluation of an mRNA vaccine against HTLV-1 using AI-driven reverse vaccinology approaches
Source: PLoS One. 2026 May 6;21(5):e0340201. doi: 10.1371/journal.pone.0340201 (PMC13148667; doi:10.1371/journal.pone.0340201)
Supplement: Attachment — Submitted filename: Response to reviewer-F2.docx [file pone.0340201.s010.docx]

Dear Editor,

Your comments and the reviewer's insights were highly insightful and enabled us to improve the quality of our manuscript greatly. The following pages include our amendments according to the journal requirements and our one-by-one responses to each of the reviewers' comments.

Revisions in the text are shown using track changes and highlights. Furthermore, the clear version of the revised manuscript (without track changes) was uploaded to the e-Component section.

1. The authors have addressed to previous suggestions and comments as well.
2. Please explain HTLV-1 epidemiology in through the world briefly; particularly, in an endemic region of Iran with scholarly references.

A brief explanation of the epidemiology and endemic areas of this virus was added to the introduction, as suggested. (page 3)

3. The whole manuscript must be revised clinically and scientifically in terms of HTLV-1 infection. For example; “The virus is also involved in other clinical disorders, such as large granular lymphocytic (LGL) leukemia, HTLV-1-associated infectious dermatitis (IDH), uveitis, and other inflammatory signs”. Therefore, the sentences should be sound for reader. Alternatively, an expert human retrovirologist/ medical virologist colleague should be useful.

We thank the reviewer for highlighting the need for clinical and scientific precision in describing HTLV-1-associated diseases. The sentence was revised to present clinical clarity. We have updated the manuscript to reflect these associations more accurately and have ensured that all clinical descriptions are consistent with current medical understanding. (page 3)

4. Please sort Keyword ascending. Please put the relevant reference(s) at the end of sentences “HTLV-1 transmission is mainly through the body's cell-containing fluids, including breast milk, blood, and semen. The virus usually exists in the intracellular form; however, its transmission is possible through direct contact”

The keywords have been alphabetically reordered as requested, and the specified reference has been added to the relevant section. (ref. 4)

5. In the introduction, for example; HTLV-1-infected patients must be divided in two categories. HTLV-1 asymptomatic carries (ACs) and/or HTLV-1 positive subjects.

As requested, the distinction between HTLV-1 asymptomatic carriers (ACs) and HTLV-1-positive subjects has been emphasized in the Introduction section by revising. (page 3)

6. Please apply “potential vaccine candidate” in the whole manuscript.

As requested, the phrase “potential vaccine candidate” has been applied consistently throughout the manuscript.

7. Far away, there is no vaccine candidate against HTLV-1 infection. Therefore, the authors have claimed mRNA vaccine strategy has prophylactic effects, while we need protective vaccine candidate against HTLV-1 infection worldwide. Therefore, the strongest strategy to HTLV-1/2 infection control and elimination is avoidance of spreading of HTLV-infection. Please explain this concern and include in the manuscript with acceptable scientific premise.

Thank you for your thoughtful feedback regarding clearly stating the position of immunization and other preventive approaches in the fight against HTLV-1. We added a paragraph to the end of the discussion section to clarify it. (pages 38 & 39) Additionally, a limitation section is included. Regarding our vaccine, the employed epitopes and predicted T-cell responses might qualify it as a candidate therapeutic vaccine too, but considering the lack of experience and vaccine in therapeutic setting, it needs a series of extensive additional evaluations and studies, so we did not focus on that.

8. Please elaborate HTLV-1 pathogenesis with scholarly references shortly.

A concise explanation of HTLV-1 pathogenesis has been added to the Introduction section and supported with scholarly references. (pages 3 & 4)

9. ACs has not needed any medication during their lifespan, please amend relevant sentences.

Relevant sentences have been added to clarify that asymptomatic carriers (ACs) typically do not require medication throughout their lifespan. (page 4)

10. In spite of HTLV-1 is RNA virus, the virus could be integrate to human chromosome and persistent during lifespan, promote oncogenesis pathways or activate chronic inflammation responses. It seems that, mRNA vaccine strategy regarding HTLV-1/2 is not effective; moreover, might be disastrous. Consequently, researchers have been focused on the subunit vaccine against pathogens. Please explain and include in the manuscript with more explanation.

We thank the reviewer for raising a critical concern regarding the suitability of mRNA vaccine platforms for HTLV-1. The question of whether mRNA vaccines are effective against HTLV-1 requires careful consideration. Importantly, mRNA vaccines do not integrate into host DNA and therefore do not share the pathogenic risks associated with retroviral integration. In principle, mRNA vaccines could elicit robust humoral and cellular immunity against HTLV-1 antigens, and the platform is well suited for inducing cytotoxic T-cell responses critical for controlling HTLV-1 infection. However, the primary challenge lies in the biology of HTLV-1 itself: because the virus persists mainly through clonal expansion of infected cells rather than ongoing virion production, vaccines must prevent initial infection rather than relying on post-exposure control. As a result, while mRNA-based approaches remain theoretically viable, current efforts continue to emphasize subunit vaccines due to their established safety profile, cost-effectiveness, and ease of antigen design within the constraints of limited HTLV-1 research funding. On the other hand, mRNA platforms offer distinct advantages in terms of safety, scalability, and rapid antigen delivery.
mRNA vaccines cannot integrate into the genome. Unlike HTLV-1, the synthetic mRNA used in vaccines:
-does not enter the nucleus
-does not undergo reverse transcription
-is rapidly degraded
-has no mechanism for integration
Therefore, mRNA vaccines do not pose a risk of contributing to HTLV-1–like integration events, and the mechanism of the virus does not make mRNA vaccination inherently dangerous. Importantly, the effectiveness of such vaccines will depend on their ability to generate sufficiently strong immune responses to block the earliest stages of infection, thereby preventing the establishment of proviral reservoirs that are otherwise resistant to immune clearance.

Accordingly, we have revised the Discussion section to include a concise clarification of the rationale for using an mRNA-based prophylactic strategy against HTLV-1. (page 35)

11. Please include HTLV-1 integrase/integrase inhibitor protein properties and its application for developing proper medication in the introduction and discussion sections.

The requested content has been carefully incorporated into both the Introduction and Discussion sections. (Pages 4 & 34-35)

12. In avoidance to Self-Citation, the authors must be removed some of previous self-cited studies.

Multiple self-citations with less importance were removed to improve the objectivity and coherence of our scientific manuscript. Considering our team’s experience in vaccine design, only a few very relevant previously published studies are kept to represent our background and experience in this field.

13. Please include the limitations of current study, before conclusion section.

We appreciate the reviewer’s suggestion and have incorporated a limitation section before the Conclusion.

I hope this modified version meets the high standards of PLOS One.

Thanks again for giving us this chance to improve our manuscript.

Best regards,

Manica Negahdaripour

Corresponding Author
